# The Dystrophin Complex Controls BK Channel Localization and Muscle Activity in *Caenorhabditis elegans*

**DOI:** 10.1371/journal.pgen.1000780

**Published:** 2009-12-18

**Authors:** Hongkyun Kim, Jonathan T. Pierce-Shimomura, Hyun J. Oh, Brandon E. Johnson, Miriam B. Goodman, Steven L. McIntire

**Affiliations:** 1 Department of Cell Biology and Anatomy, The Chicago Medical School, Rosalind Franklin University of Science and Medicine, North Chicago, Illinois, United States of America; 2 Ernest Gallo Clinic and Research Center, Department of Neurology, University of California San Francisco, Emeryville, California, United States of America; 3 Department of Molecular and Cellular Physiology, Stanford University School of Medicine, Stanford, California, United States of America; The University of North Carolina at Chapel Hill, United States of America

## Abstract

Genetic defects in the dystrophin-associated protein complex (DAPC) are responsible for a variety of pathological conditions including muscular dystrophy, cardiomyopathy, and vasospasm. Conserved DAPC components from humans to *Caenorhabditis elegans* suggest a similar molecular function. *C. elegans* DAPC mutants exhibit a unique locomotory deficit resulting from prolonged muscle excitation and contraction. Here we show that the *C. elegans* DAPC is essential for proper localization of SLO-1, the large conductance, voltage-, and calcium-dependent potassium (BK) channel, which conducts a major outward rectifying current in muscle under the normal physiological condition. Through analysis of mutants with the same phenotype as the DAPC mutants, we identified the novel *islo-1* gene that encodes a protein with two predicted transmembrane domains. We demonstrate that ISLO-1 acts as a novel adapter molecule that links the DAPC to SLO-1 in muscle. We show that a defect in either the DAPC or ISLO-1 disrupts normal SLO-1 localization in muscle. Consistent with observations that SLO-1 requires a high calcium concentration for full activation, we find that SLO-1 is localized near L-type calcium channels in muscle, thereby providing a mechanism coupling calcium influx with the outward rectifying current. Our results indicate that the DAPC modulates muscle excitability by localizing the SLO-1 channel to calcium-rich regions of *C. elegans* muscle.

## Author Summary

Dystrophin is a long rod-shaped protein that forms a complex with several membrane and cytoplasmic proteins in muscle. Genetic defects in components of this dystrophin complex are responsible for many forms of muscular dystrophy, including Duchenne muscular dystrophy. *C. elegans* possesses the dystrophin complex and mutations in its components cause muscular defects, indicating that the dystrophin complex has an evolutionary conserved role in muscle. Accumulating evidence in mammals indicates that dystrophic muscle exhibits an abnormal calcium homeostasis. It is not clear how defects in the dystrophin complex are linked to calcium homeostasis, however. In a *C. elegans* genetic study we found that a novel adaptor protein links the dystrophin complex to a calcium-sensitive potassium channel that mediates muscle inactivation. We further demonstrated that both the dystrophin complex and the adaptor protein localize the potassium channel in a close proximity to a muscle-activating calcium channel. This arrangement ensures that calcium increases accompanied by muscle activation are coupled to muscle inactivation. Defects in the dystrophin complex or the adaptor disrupt the localization of the potassium channel, thereby resulting in prolonged muscle activation and calcium ion increases. Our study provides a mechanism by which the dystrophin complex regulates cellular signaling and muscle excitability.

## Introduction

The dystrophin-associated protein complex (DAPC) is a multimeric protein complex found in the muscle membrane that has been implicated in many degenerative or pathological conditions, including muscular dystrophy, vasospasm and cardiomyopathy [1],[2]. Elucidation of the molecular function of the DAPC will increase our understanding of the pathogenesis of these conditions. Studies indicate that the DAPC contributes to the structural stability of muscle cells by forming a link between the cytoskeleton and the extracellular matrix and functions as a scaffolding complex for signaling molecules [1]. Several lines of evidence indicate that lack of dystrophin results in elevated intracellular calcium levels [3] and altered calcium-mediated muscle contractility [4],[5]. The involvement of the DAPC in calcium-mediated responses is particularly intriguing, given that the abnormal regulation of calcium ions has been linked to necrotic cell death and excitotoxic injury. Although it is clear that dystrophic muscle exhibits abnormal calcium homeostasis and has elevated calcium levels, there is some controversy concerning which ion channels or pumps mediate such calcium increases. Several calcium-permeable ion channels in the muscle membrane, including L-type calcium channels and TRP channels, have been suggested to underlie an intracellular calcium increase in dystrophic muscle [6]. On the other hand, other studies suggest that defects in dystrophin weaken the tight regulation of calcium release from the sarcoplasmic reticulum [4],[7]. In this case, elevated calcium concentrations may originate from a persistent leak from calcium stores or activation of store-operated channels [7]. These controversial results may be explained in part by different experimental settings or sample preparations. It is also possible that some of these channels mediate calcium increases in parallel, or that one of them disrupts calcium homeostasis by altering another calcium channel or pump. Regardless, none of these prior studies directly link defects in the DAPC to ion channel dysfunction.

*C. elegans* is the simplest model organism that possesses most DAPC components and mutations in DAPC genes of *C. elegans* cause unique phenotypes, including exaggerated head-bending and hypercontraction[8],[9]. We previously described a forward genetic screen to identify genes encoding additional components of the DAPC or genes that function in the same biochemical or physiological pathway as DAPC genes [10]. Here, we identify a novel DAPC-interacting protein, ISLO-1. We show that ISLO-1 acts as an adapter molecule by physically interacting with SLO-1 (the ortholog of the mammalian BK channel) as well as the DAPC. Interaction of SLO-1 with the DAPC through ISLO-1 serves to localize the SLO-1 channel near L-type calcium channels. SLO-1 channels are activated by calcium influx and the resulting potassium efflux negatively regulates the calcium-mediated activation of muscle. We show that a compromise in either the DAPC or ISLO-1 abrogates normal SLO-1 channel localization and results in excessive muscle contraction. These results provide a molecular mechanism by which the DAPC may regulate calcium-mediated responses and protect muscles from overexcitation and consequent cellular damage.

## Results

### *islo-1* functions in the same pathway as *slo-1* and the DAPC genes

**Figure 1:**
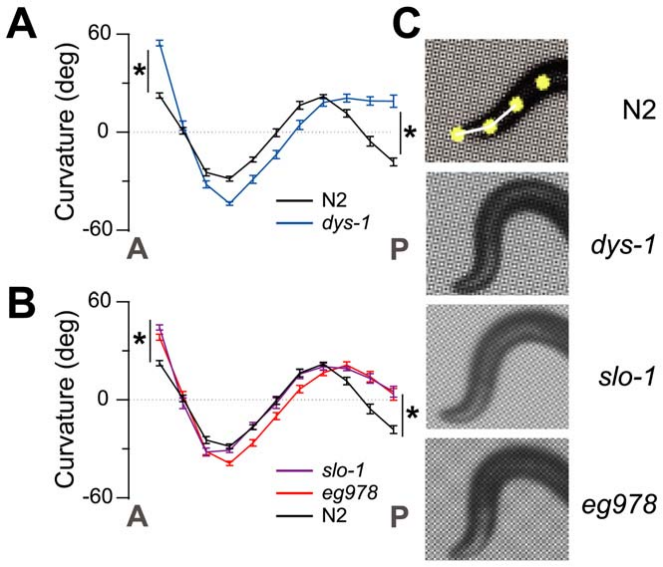
Locomotion analysis of wild-type and head-bending mutant animals. (A,B) Plots of average curvature values (angles) versus body position (11 positions) arranged anterior (A) to posterior (P). Asterisks indicate significantly deeper head bending than wild type (N2) for the first and 11th angles (*P*<0.05). We also observed significant difference in 11th angles between *dys-1* and *islo-1* (P<0.01). This difference may result from the phenotypic severity between *dys-1* and *islo-1* mutants. The genotypes of mutant animals are as follows: *dys-1(eg33)*, *slo-1(eg111)* and *islo-1(eg978)*. Error bars, SEM. (C) Representative photos show the anterior portion of wild-type and mutant animals. Yellow dots indicate the four most anterior of the 13 midline points for a wild-type animal. The first curvature angle is indicated in two white line segments.

Prior qualitative studies of the crawling pattern of *C. elegans* DAPC mutants have suggested that they exhibit characteristic locomotory phenotypes, including periodic hypercontraction and excessive head bending during forward movement [8],[11],[12]. By quantifying the posture of crawling animals with an image analysis system [13], we found that *dys-1* (the *C. elegans* dystrophin ortholog) mutants display an unusual degree of excessive bending at the anterior tip of the body (Figure 1). We have referred to this unique aspect of the DAPC locomotory phenotype as the head-bending phenotype [9]. A previous study[14] also revealed that the locomotory phenotype of the *slo-1* mutant resembles that of DAPC mutants. Consistent with this observation, we quantified head-bending of a *slo-1* mutant and found that it was indistinguishable from a *dys-1* mutant (Figure 1). *slo-1* encodes a calcium- and voltage-dependent potassium (BK) channel [15].

**Figure 2:**
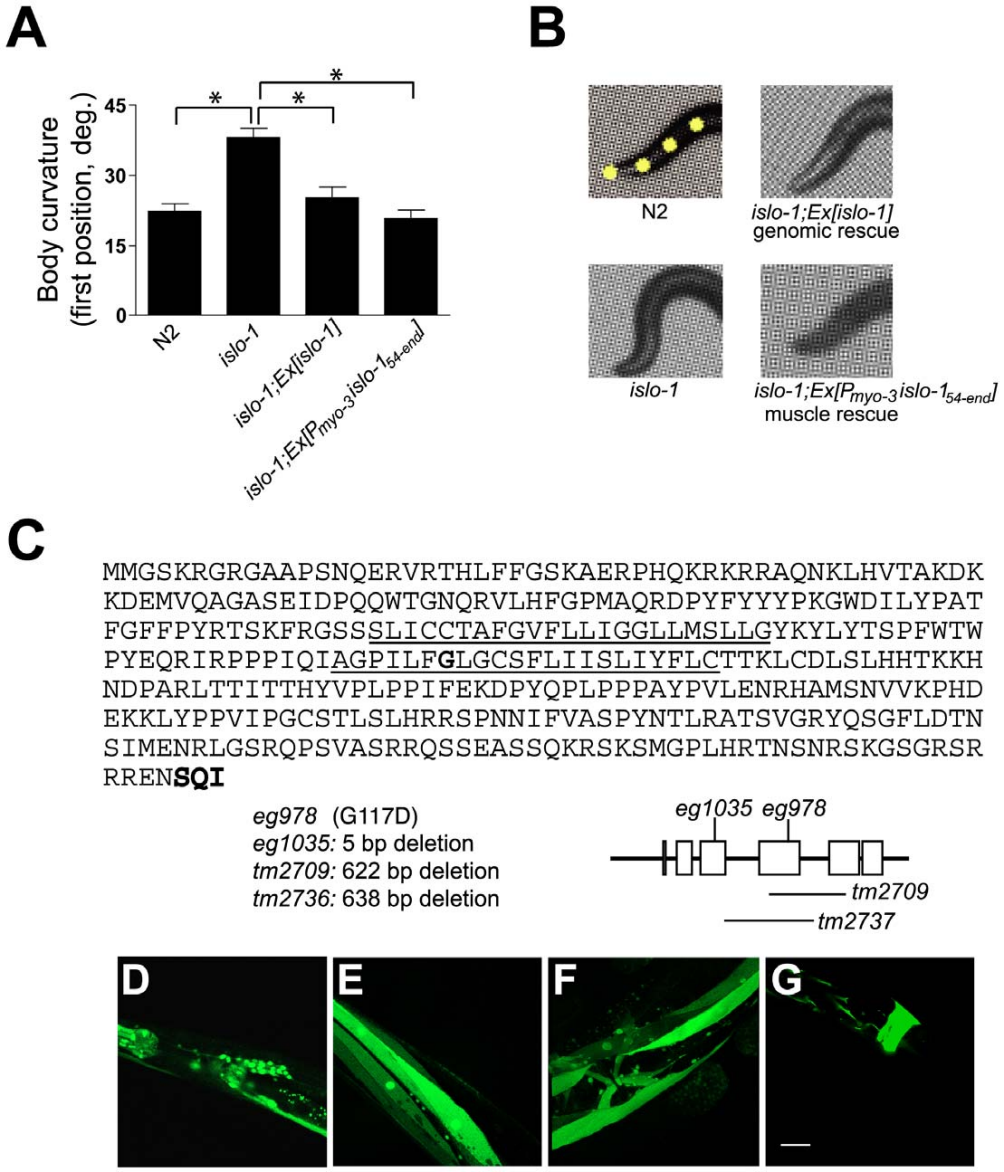
Transgenic rescue and cloning of *islo-1*. (A) The first curvature angle obtained from wild-type (N2) and *islo-1(eg978)*, and *islo-1* transgenic rescue strains (genomic rescue strain, *islo-1*;*Ex*[*islo-1*], and muscle specific rescue strain, *islo-1*;Ex[P *_myo-3_ islo-1_54-end_*]). Error bars, SEM. Asterisks represent significant difference between *islo-1* and other animal groups (*P*<0.05). (B) Representative photos show the anterior portion of animals. (C) The predicted amino acid sequence of ISLO-1. The predicted transmembrane domain sequences are underlined and the type I PDZ recognition sequence is bold. (D–G) The expression pattern of the *islo-1* gene. The upstream sequence of the predicted translation initiation site was inserted into a GFP coding sequence in frame, and the resulting DNA was used for generating transgenic animals in wild-type animals. Expression was observed in neurons (D), body-wall muscles (E), egg-laying muscles (F) and enteric muscles (G). Scale bar, 10 µm.

We obtained a novel mutant, *eg978*, that shares the head-bending phenotype of *dys-1* and *slo-1* from a forward genetic screen that identified *slo-1* and many DAPC mutants (Figure 1). Our quantitative analysis demonstrates that the head-bending phenotype is shared between DAPC mutants and the *slo-1* and *eg978* mutants. The shared head-bending phenotype of DAPC, *slo-1* and *eg978* mutants suggests a related function for these genes. The phenotype of the double *eg978;dys-1* mutant was not more severe than that of *dys-1* mutants (Figure S1), providing an additional support for the idea that two genes function in the same genetic pathway. To better understand how *eg978* relates to other DAPC components, we cloned the gene by genetic mapping and transgenic rescue (Figure S2). We mapped *eg978* to the middle of chromosome IV by SNP (single nucleotide polymorphism) mapping based on the head-bending phenotype, and rescued the phenotype with a single cosmid, F42G8 (Figure 2). A PCR fragment containing a single open reading frame, F42G8.5, rescued the phenotype. We determined the predicted coding sequence of *eg978* and found that a glycine in the second transmembrane domain was changed to aspartic acid. We examined other deletion alleles and found that they show an identical phenotype (Figure S1), suggesting that all of these mutations cause a null or strong loss-of-function phenotype. We named F42G8.5 *islo-1* (*i*nteractor with *slo-1*; see below). The predicted *islo-1* gene encodes a protein with two predicted transmembrane domains and a type I PDZ recognition sequence that interacts with the PDZ domain of syntrophin (STN-1), a known component of the DAPC (Figure 2). Interestingly, *islo-1* is transcribed as part of an operon with *pmk-3*, *-2* and *-1* genes, which are homologues of stress-activated mitogen-activated p38 kinases [16]. Consistent with the previously reported expression pattern of the *pmk-3* gene [16], we observed neuronal expression with an *islo-1* promoter-tagged GFP reporter. Additionally, we observed expression throughout all muscle tissues, including body wall, pharyngeal, enteric and egg-laying muscles (Figure 2D–2G). All of the observed phenotypes could be rescued by muscle-specific expression of *islo-1*, indicating that the head-bending phenotype results from a muscular defect (Figure 2A and 2B).

### ISLO-1 interacts with the dystrophin complex

**Figure 3:**
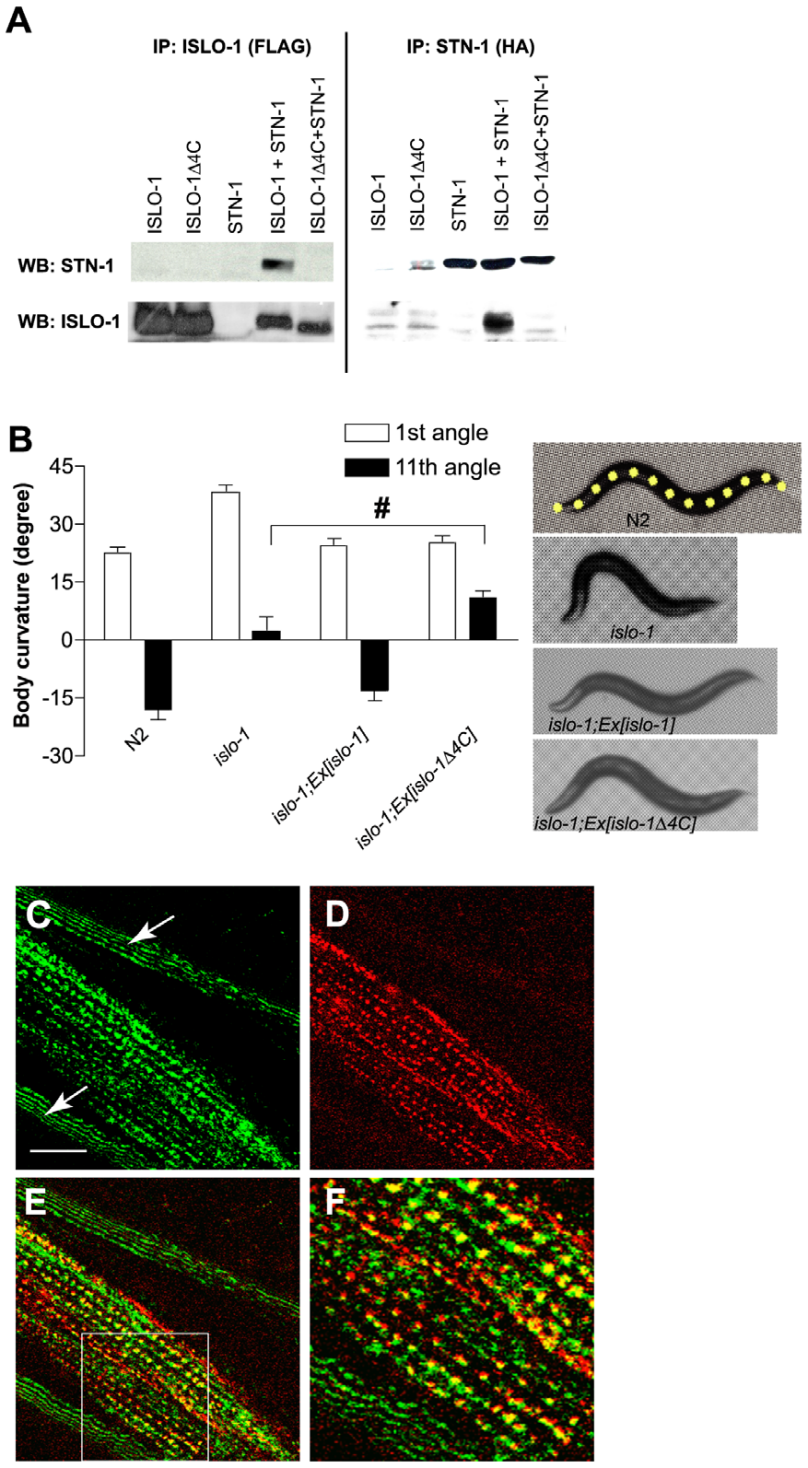
ISLO-1 interacts with the dystrophin complex through STN-1. (A) Co-Immunoprecipitation of ISLO-1_54-end_ and STN-1. ISLO-1_54-end_ (or ISLO-1Δ4C_54-354_ lacking the concensus type I PDZ interaction sequence) and STN-1 were tagged with FLAG and HA epitope, respectively. These constructs were used for transient transfection in HEK 293 cells. Immunoprecipitation (IP) and Western blot analysis (WB) was performed with FLAG or HA antibody. (B) The first and eleventh curvature angles obtained from wild-type, *islo-1* and *islo-1* transgenic animals. For the first angle, N2 versus *islo-1*, *islo-1* versus *islo-1*;Ex[*islo-1*] and *islo-1* versus *islo-1*;Ex[*islo-1*Δ4C] are significantly different from each other (P<0.05). For the 11^th^ angle, N2 versus *islo-1* and *islo-1* versus *islo-1*;Ex[*islo-1*] are significantly different from each other (P<0.05). Number sign indicates no significant difference from *islo-1* for tail angle value. Error bars, SEM. Representative photos show whole-body posture of animals on right including the 13 yellow midline points superimposed on the image of a wild-type animal. (C–F) Integrated transgenes of GFP-tagged *sgca-1* (C) and mCherry-tagged *islo-1* (D) are shown in the *islo-1*;*sgca-1* double mutant. Arrows denote the edge of cuticle. (E) Merged image of C and D. (F) Enlarged image of inset in E.

Because *islo-1* mutants exhibit a head-bending phenotype indistinguishable from DAPC mutants and its predicted coding sequence has a type I PDZ recognition sequence, we hypothesized that the ISLO-1 protein associates with the DAPC through PDZ dependent interaction with STN-1. To test this hypothesis, we transfected epitope-tagged ISLO-1 and STN-1 into HEK293 cells, and performed co-immunopreciptiation experiments. We observed a reciprocal *in vitro* interaction between ISLO-1 and STN-1, but not between ISLO-1 lacking the PDZ recognition motif and STN-1 (Figure 3A). We then evaluated the biological significance of this *in vitro* interaction by introducing an *islo-1* construct with a deleted PDZ recognition motif into *islo-1* mutants. This mutant *islo-1* transgene failed to rescue the head-bending phenotype completely (4 of 4 stable lines), indicating that the PDZ interaction is necessary for proper function (Figure 3B).

We further examined the subcellular localization of the ISLO-1 protein with mCherry-tagged *islo-1*, which could rescue the locomotory phenotype (Figure S1). We observed punctate structures in muscle membrane, reminiscent of the *slo-1* expression pattern (Figure 3D) [15]. We then assessed whether the ISLO-1 protein localizes with the DAPC *in vivo* by tagging SGCA (an α-sarcoglyan homologue that forms part of the DAPC) with GFP. GFP::SGCA-1 showed a punctate pattern throughout the muscle membrane (Figure 3C). Furthermore, GFP puncta were enriched where ISLO-1 is localized, indicating that SGCA-1 is localized in close proximity to ISLO-1 (Figure 3C–3F). Taken together, these results indicate that ISLO-1 is associated with the DAPC through the PDZ domain of STN-1.

To identify the punctate structure where the DAPC and ISLO-1 are localized, we stained wild-type animals with SGCA-1 antibodies, together with antibodies that recognize a myosin heavy chain (A band) or vinculin (dense bodies, analogous to Z line/costamere). SGCA-1 staining was excluded from the A band region. Instead SGCA-1 was partially co-localized with vinculin only in dense bodies, indicating that the DAPC is localized near dense bodies (Figure S3).

### ISLO-1 mediates the interaction of SLO-1 with the dystrophin complex

**Figure 4:**
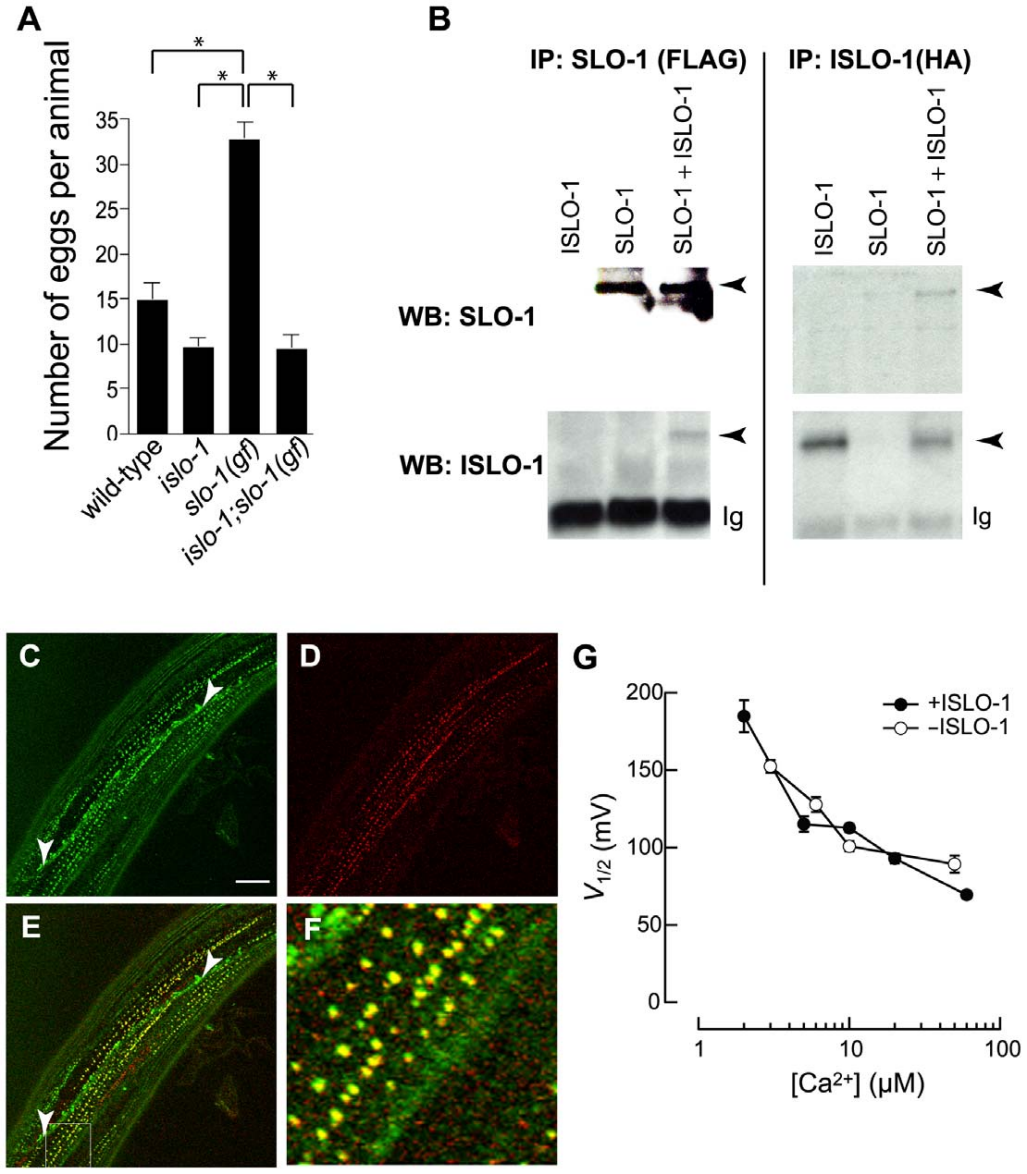
ISLO-1 represents a novel subunit of SLO-1 channel. (A) The number of eggs retained in uteri was counted for each of the indicated mutants 30 hrs after the L4 stage. Error bars, SEM. Asterisks represent significant difference from each other (*P*<0.0001) (B) Co-immunoprecipitation of SLO-1 and ISLO-1_54-end_. SLO-1 and ISLO-1_54-end_ are tagged with FLAG and HA epitope at the N-termini, respectively. Immunoprecipitations and western blot analysis were performed as Figure 3A. Black arrowheads represent specific bands. Ig, immunoglobulin light chain. (C–F) Integrated transgenes of a GFP-tagged *slo-1* (C) and a mCherry-tagged *islo-1* (D) are shown in the *islo-1*;*slo-1* double mutant. White arrowheads denote the neuronal processes where co-localization is not observed. (E) Merged image of (C,D). (F) Enlarged image of inset in E. (G) *V*_1/2_ as a function of [Ca^2+^]_i_ was derived from macroscopic G-V curves (not shown). Filled (*n* = 3) and open circles (*n* = 5) are the average values in the presence and absence of ISLO-1, respectively. Some SEM error bars are smaller than the symbols.

Our previous genetic screen identified mutations in DAPC genes [12]. This screen also identified many alleles of the calcium- and voltage-dependent BK potassium channel gene *slo-1* [10]. Carre-Pierrat *et al.* [14] noted that the locomotory phenotype of *slo-1* mutants qualitatively resembles that of DAPC mutants, and that this phenotype is rescued by muscular expression of *slo-1*. These results led them to suggest that *slo-1* function may be regulated by the DAPC. Intrigued by this possibility, we explored whether *islo-1* interacts with *slo-1*. First, we examined a possible genetic interaction between *slo-1* and *islo-1*. A gain-of-function *slo-1* mutation (*ky399gf*) causes an increase in open probability of the SLO-1 channel *in vivo* due to changes in activation and deactivation kinetics [10]. Such an increase would result in the hyperpolarization of muscles, including egg-laying muscle. *slo-1*(*ky399gf*) mutants are defective in egg-laying and retain twice as many eggs as wild-type animals (Figure 4A). If *islo-1* is important for the function of *slo-1*, then we predicted that an *islo-1* mutation should suppress a *slo-1*(*gf*) phenotype. We observed that the *islo-1* mutation suppresses the egg-laying defect of *slo-1*(*gf*) mutants, suggesting that *islo-1* may regulate *slo-1* function.

This genetic interaction prompted us to examine whether the SLO-1 channel interacts physically with ISLO-1 using co-immunoprecipitation (Figure 4B). In transiently transfected HEK293 cells, immunoprecipitation of SLO-1 brought down ISLO-1. Reciprocally, immunoprecipitation of ISLO-1 brought down SLO-1. These results indicate that the SLO-1 channel and ISLO-1 protein are physically associated with each other *in vitro*. Finally, we examined whether the SLO-1 channel and ISLO-1 protein are co-localized in the muscle membrane. GFP- tagged SLO-1 and mCherry-tagged ISLO-1 were utilized to generate transgenic animals in the *islo-1;slo-1* double mutant (Figure 4C–4F). This GFP-tagged SLO-1 construct was successfully used for examining SLO-1 localization [15] and the mCherry-tagged ISLO-1 construct can rescue the phenotype (Figure S1). Consistent with previous observations, SLO-1 exhibited a punctate pattern in muscle membrane [15]. Similarly, we found that ISLO-1::mCherry also exhibited a punctate pattern in muscle membrane. Furthermore, these two punctate patterns overlapped on the muscle membrane in the same plane on confocal imaging, indicating that SLO-1 and ISLO-1 are colocalized. Together, our genetic, biochemical and cell biological data indicate that the SLO-1 channel interacts with the dystrophin complex through the ISLO-1 protein in body wall muscle.

**Figure 5:**
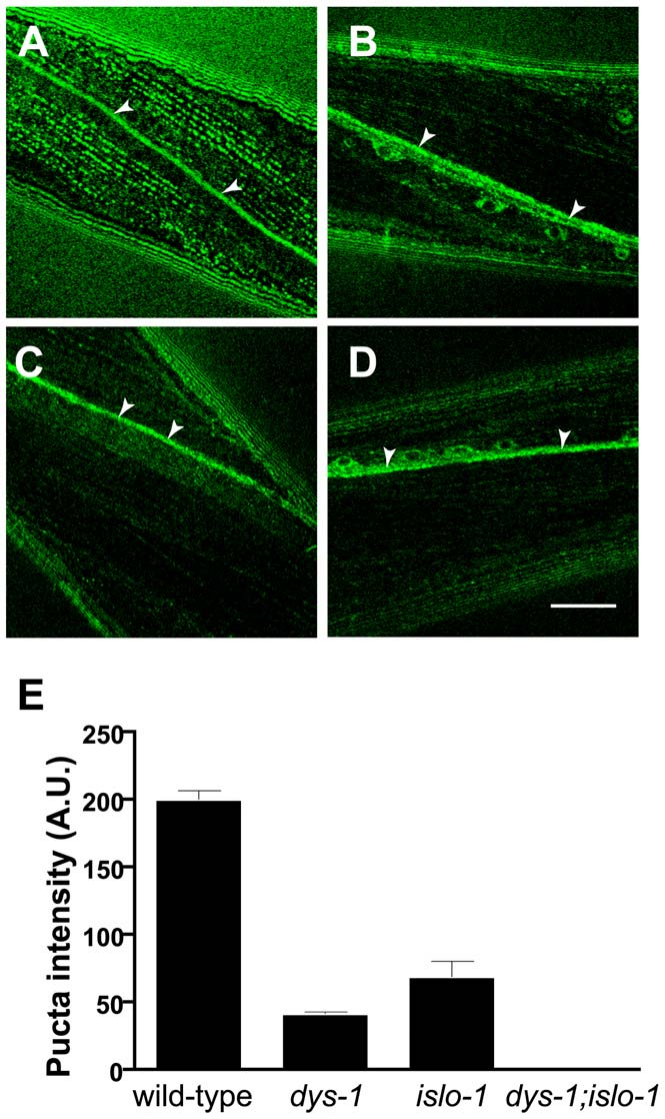
The dystrophin complex is necessary for localization of the SLO-1 channel in muscles, but not in neurons. (A–D) The localization of SLO-1::GFP in *wild-type* (A), *dys-1* (B), *islo-1* (C), and *islo-1;dys-1* (D). The same integrated array, SLO-1::GFP, was used for this analysis in different genetic backgrounds. To make the expression level of SLO-1::GFP close to *in vivo*, we introduced a *slo-1* loss-of-function mutation to all of strains used in this experiment. Arrowheads indicate SLO-1::GFP localized in neurons. Scale bar, 10 µm. (E) The quantification analysis of puncta intensity in muscle membrane. The intensity of puncta was analyzed as described in Materials and Methods. At least 50 animals from each genotype were observed. Error bars, SEM. *P* values (unpaired T test) for comparison between the wild-type animal group and other animal groups are below 0.001.

After establishing a physical interaction between SLO-1 and ISLO-1, we examined whether ISLO-1 alters the voltage or calcium dependence of SLO-1 currents by co-expressing both proteins in Xenopus oocytes. We found that ISLO-1 had no detectable effect on either aspect of channel function (Figure 4G). While it is possible this reflects a failure of ISLO-1 to express in oocytes, this seems unlikely given that the same protein can be expressed in mammalian cells (see Figure 3B). What then is the significance of the interaction between SLO-1 and ISLO-1? One possibility is that ISLO-1 and the dystrophin complex localize SLO-1 to a specific cellular environment. If this is the case, then the localization of SLO-1 will be compromised in mutants that are defective in either the dystrophin complex or *islo-1*. We examined whether the subcellular localization of the GFP-tagged SLO-1 [15] was perturbed in *dys-1* and *islo-1* single mutants and *dys-1*;*islo-1* double mutants (Figure 5A–5D). The intensity of SLO-1::GFP puncta in the muscle membrane was greatly diminished in *dys-1*or *islo-1* single mutant backgrounds (Figure 5B, 5C, and 5E), and puncta could not be detected in *dys-1*;*islo-1* double mutants (Figure 5D and 5E). Interestingly, the neural expression of *slo-1* appeared identical in all mutant backgrounds. Given that SLO-1:: GFP localization was greatly reduced but not completely abolished in *dys-1* or *islo-1* single mutants, it is possible that the DAPC and ISLO-1 are both essential for maintaining or stabilizing SLO-1 localization near dense bodies. Together, these results strongly suggest that the dystrophin complex and ISLO-1 are necessary for maintaining proper localization of SLO-1 in muscle membrane.

### The dystrophin complex and ISLO-1 regulate localization of BK channels

**Figure 6:**
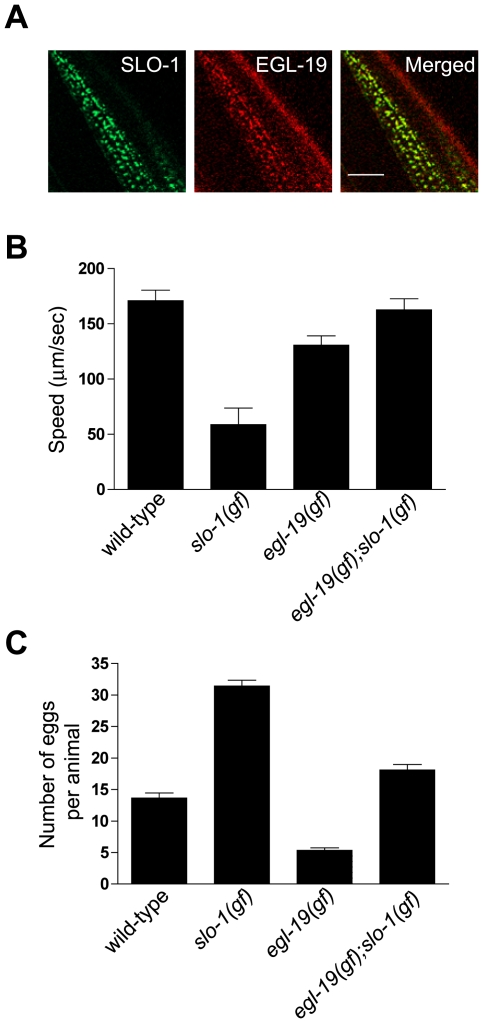
The SLO-1 channel and the L-type EGL-19 calcium channel functionally interact with each other. (A) Transgenic animals with integrated SLO-1::GFP and extrachromosomal array EGL-19::mCherry were used for localizing SLO-1 and EGL-19. The *right* side image shows a merged image. Scale bar, 10 µm. Analysis of locomotion (B) and retained eggs (C) in wild-type, *egl-19(gf)*, *slo-1(gf)* and *egl-19(gf);slo-1(gf)* mutant animals. Approximately ten animals of the same genotype were tested for both of phenotypes. Error bars, SEM. Statistical significance was determined by two-tailed unpaired T test. Respective P values for locomotion and egg laying are: *egl-19(gf)* versus *egl-19(gf);slo-1(gf)*, *P<0.05*, *P<0.0001*; *slo-1(gf)* versus *egl-19(gf);slo-1(gf)*, *P<0.0001*, *P<0.0001*; wild-type versus *egl-19(gf);slo-1(gf)*, *P*>0.05, *P*<0.05.

Carre-Pierrat *et al.* [14] found that the SLO-1 channel shows indistinguishable single-channel conductance and kinetics from recordings of excised patches from wild-type and *dys-1* mutant muscle. These *in vivo* results and our finding that ISLO-1 has no detectable effect on voltage- or calcium-sensitivity of BK channels expressed in heterologous cells (Figure 4G) argue against the possibility that the dystrophin complex and ISLO-1 modify the channel properties of SLO-1 directly [14]. Another intriguing possibility is that the dystrophin complex and ISLO-1 may affect channel activity by regulating the localization of SLO-1. The activation of the SLO-1 channel in mammals and *C. elegans* requires high concentrations of calcium ions (>10 µM) [15],[17],[18]. Such high levels of calcium ions are tightly restricted in time and space to specific subcellular areas where calcium channels are localized [19],[20]. In mammals, it has been suggested that BK channels in other tissues may be in close proximity to calcium channels, such as L-type calcium channels [21],[22]. The dystrophin complex and ISLO-1 can promote the localization of the SLO-1 channel to calcium-rich regions where SLO-1 can play a role in preventing hyper-excitation and hyper-contraction of muscle in response to large calcium increases. Therefore, we examined whether the SLO-1 channel is localized near the *C. elegans* homologue of the L-type calcium channel (EGL-19) by using GTP-tagged SLO-1 and mCherry-tagged EGL-19. EGL-19::mCherry fluorescence shows a punctate pattern on the surface of the muscle membrane (Figure 6B). Furthermore, these EGL-19::mCherry puncta are co-localized with SLO-1:GFP puncta (Figure 6C). Interestingly, most, but not all, of SLO-1::GFP puncta are co-localized with EGL-19::mCherry puncta.

The co-localization of EGL-19 with SLO-1 channels led us to test for a genetic interaction between *egl-19* and *slo-1*. *egl-19(* *n2368gf)* gain-of-function mutants exhibit an increased frequency and duration of egg-laying muscle contraction, and show a reduced body length likely due to muscle hypercontraction [23]. This phenotype is consistent with increased calcium entry in *egl-19(gf)* mutants. If EGL-19 and SLO-1 channels functionally interact with each other, then a *slo-1(* *ky399gf)* gain-of-function mutation should suppress the *egl-19(gf)* phenotype. Because the gating of SLO-1 channels is calcium-dependent and the *slo-1(ky399gf)* mutation causes delayed closing kinetics, we reasoned that the *slo-1(gf)* mutation may offset the activating effect of *egl-19(gf)* on muscle. Indeed, the locomotion of double mutants, *egl-19(* *gf)*;*slo-1(* *ky399gf)*, was dramatically improved when compared with those of single mutants alone (Figure 6B, see Video S1). Furthermore, the number of eggs retained by double mutant animals was different from that of single *slo-1(* *ky399gf)* or *egl-19(gf)* mutants and was similar to the level of wild-type animals (Figure 6C). Together, these results support the notion that the localization of SLO-1 near L-type Ca^2+^ channels where calcium entry gives rise to high local concentrations is essential for proper SLO-1 function, and that the potassium current mediated by SLO-1 attenuates calcium-dependent excitation of muscle. The functional coupling of SLO-1 channels with L-type Ca^2+^ channels may prevent muscle overexcitation or overstimulation. Defects in the dystrophin complex and ISLO-1 disrupt the localization and thus function of the SLO-1 channels, providing an explanation for the hypercontraction of muscles and exaggerated head bending in the DAPC mutants.

## Discussion

In this study we identified the novel *islo-1* gene and showed that it functions in the same pathway as *slo-1* and DAPC genes. The *islo-1* gene encodes a protein that has two transmembrane domains with a type I PDZ interaction domain at the C-terminal end, although it does not show an obvious homology with any other known protein. Previously, Carre-Pierrat *et al.* [14] showed that *slo-1* genetically interacts with DAPC genes, and suggested that SLO-1 may directly or indirectly interact with the DAPC. In our study we found that the newly-identified ISLO-1 interacts with the SLO-1 channels in muscle. In this sense, ISLO-1 is analogous to mammalian auxiliary beta subunits of BK channels that also consist of two transmembrane domains. These auxiliary beta subunits ( β1, β2, β3 and β4) are expressed in a tissue specific manner [24]. For instance, β1 is prominently expressed in smooth muscle and β4 is highly expressed in brain. Interestingly, no beta subunit expressed in skeletal muscle has been identified so far, suggesting that skeletal muscle may lack a BK channel beta subunit or may possess a divergent beta subunit. All of the known beta subunits influence channel properties such as activation time course and voltage-dependence. β1 also controls the localization and surface expression of the BK channels [25]. Our data indicate that, unlike mammalian auxiliary beta subunits, the function of ISLO-1 is restricted to controlling the localization of the SLO-1 channels. ISLO-1 controls SLO-1 localization by tethering them to the DAPC. Hence, defects in ISLO-1 or the DAPC result in disruption of normal localization of SLO-1.

Despite the critical role of EGL-19 in muscle activation, its exact localization in the muscle membrane has not been determined previously [26]. Likewise, while the *C. elegans* DAPC is important for localizing signaling molecules and possibly providing structural stability in the muscle membrane, its localization in the muscle membrane has not been defined until now. In this study we identified the localization of these two important molecules in body wall muscle cells. Interestingly, EGL-19 localization overlaps with SLO-1 near the dense bodies (equivalent to mammalian costameres). In mammals, SLO-1 and a subtype of L-type Ca^2+^channels (Cav1.2) can be co-purified together and form a complex that reconstitutes calcium nanodomains [22]. We showed that the *egl-19(gf);slo-1(gf)* double mutants move faster than single *egl-19(gf)* and *slo-1(gf)* mutants (Figure 6C). Notably, we observed that mutations in DAPC genes that disrupt SLO-1 localization do not convert the phenotype of *egl-19* gain-of-function mutants to the loss-of-function phenotype (unpublished data, HK). This observation suggests that SLO-1 and the DAPC do not directly control the localization of EGL-19. Although we do not know whether or not SLO-1 and EGL-19 physically interact with each other, EGL-19 localization is likely to be controlled by a distinct set of molecules.

Based on our results, we conclude that the head-bending phenotype of DAPC mutants likely results from muscle hypercontration caused by a defect in feedback inhibition by the BK channels. Hence, abnormal calcium increases in DAPC mutants probably result from aberrant muscle excitation and contraction process, which involves the L-type Ca^2+^ channels. In fact, there is ample evidence that abnormal calcium increases in mammalian dystrophic muscle are mediated through excitation-contraction coupling, where the L-type Ca^2+^ channel plays an essential role. First, dystrophin is also localized near L-type Ca^2+^ channels in skeletal muscle [27]. Previous studies with fixed skeletal muscle showed the enrichment of the DAPC near the costamere, but not in the triad region [28]. A confocal immunofluorescence staining without fixing, however, showed that dystrophin is also found in the triad regions where excitation-contraction coupling takes place and the BK channels and the L-type Ca^2+^ channels are localized [27]. Second, there is direct evidence that calcium homeostasis is disrupted in the triad regions of dystrophic muscle. Calcium sparks, a highly localized calcium transients originating at the triad, are mediated by ryanodine receptors and L-type Ca^2+^ channels and cause BK channel activation, which is responsible for muscle inactivation. Skeletal muscle from *mdx* mice, a rodent model of Duchenne muscular dystrophy, exhibits an abnormally high frequency and amplitude of calcium sparks [4]. Third, vasospasm (constriction of blood vessel smooth muscle) is observed in *mdx* mice and γ- or δ- sarcoglycan deficient mice [29],[30] and this vasospasm is relieved by verapamil, an L-type calcium channel blocker [30]. Interestingly, BK channels negatively regulate tone in vascular smooth muscle and increased BK channel expression protects from vasospasm [31]. Lastly, delayed inactivation of L-type calcium channels was observed in *mdx* mouse muscle [32]. This could lead to the prolonged muscle activation and calcium increases. Interestingly, a single twitch contraction of skeletal muscle does not depend on extracellular calcium ions. Tetanic muscle activations triggered by high frequency of action potentials, however, are dependent on extracellular calcium influx through L-type Ca^2+^ channels [33]. Perhaps during periods of tetanic contraction, calcium influx through the L-type Ca^2+^ channels is particularly important for activation of BK channels and terminating contraction. Consistent with this theory, we previously showed that the locomotory phenotypes of *C. elegans* DAPC mutants become obvious when animals show a rapid escape response [9].

How does the connection of the DAPC with the BK channel contribute to the pathogenesis of muscular dystrophy? Given the functional coupling between the BK channel and the L-type Ca^2+^ channel in *C. elegans* and mammalian muscle, we propose that the tight coupling of high calcium influx to the outward rectifying potassium current protects muscle from overexcitation and potential cellular damage. A defect in the DAPC disrupts coupling of the BK channel to L-type Ca^2+^ channels, causing a prolonged muscle activation and elevated calcium ions. It has been postulated that elevated intracellular calcium ions in muscle causes activation of Ca^2+^ -dependent proteases and subsequently facilitates necrosis [34]. Our current study provides a mechanism by which the DAPC regulates cellular signaling and muscle excitability.

## Materials and Methods

### Strains

Strains were maintained by standard methods [35]. The genotypes of animals used in this study are: N2 (wild-type), CB4856, *dys-1(eg33) I*, *stn-1(tm795) I*, *egl-19(n2368) IV*, *egl-19(n582) IV*, *egl-19*(*st556*)/*unc-82*(*e1323*) *unc-24*(*e138*) *IV*, *islo-1(eg978) IV*, *islo-1(eg1035) IV*, *islo-1(tm2709) IV*, *islo-1(tm2736) IV*, *slo-1(eg142) V*, *slo-1(eg111) V*, *slo-1(ky399) V* and *sgca-1(tm1232) X*. The following transgenes were used in this study: *Is[slo-1a::GFP, rol-6(d)]*, *Is[GFP::islo-1, rol-6(d)]*, *Is[mCherry::islo-1, GFP::sgca-1, rol-6(d)]*, *Is[mCherry::islo-1, slo-1::GFP, rol-6(d)]*, *Ex[Pmyo-3GFP::islo-1_54-end_, ofm-1::GFP]*, *Ex[islo-1, ofm-1::GFP]*, *Ex[islo-1Δ4C, ofm-1::GFP]* and *Ex*[*egl-19::mCherry*] (details in Text S1). The muscle specific *islo-1* construct lacks 53 amino acids at the amino terminus, but rescues all phenotypes as well as with the full-length genomic construct. Hence, some experiments were performed with the shorter form (indicated as *islo-1*_54-end_). To observe changes in SLO-1::GFP fluorescence of animals with different genotypes, we used the same integrated transgene, *Is[slo-1::GFP, rol-6(d)]*.

### Genetic mapping and cloning

Based on the head-bending phenotype we mapped *eg978* by analyzing single nucleotide polymorphisms present between N2 and CB4856 strains [36]. The corresponding gene was identified by transgenic rescue and confirmed by sequence analysis.

### Immunolocalization and fluorescent microscopy

The peptide sequence close to the C terminus (CRNPLSNESDEQDTE) of SGCA-1 was targeted for raising rabbit polyclonal anti-SGCA-1 antibody. Staining of *sgca-1* deletion mutants with this antibody did not show any detectable muscle structure, demonstrating the specificity of affinity purified anti-SGCA-1 antibody. Fixation and immunostaining procedures are described in Text S1 (Supplementary Methods). Animals expressing GFP/mCherry-tagged transgenes or immunostained samples were observed under a Zeiss laser scanning confocal microscope (LSM 510 meta) with a 63x oil-immersion objective (numerical aperture: 1.4) or an Olympus Fluoview 300 with a 60x oil-immersion objective (numerical aperture: 1.4). When two channels were used, images are acquired sequentially with the pinhole diameter set to 1 (for immunostaining) or 1.2 (for GFP/mCherry) Airy units. For comparison of fluorescence intensities, images were acquired under an identical exposure time, gain and pinhole diameter. The intensity of puncta was analyzed using linescan (Metamorph, Molecular Devices) and presented as values obtained by subtracting background levels from averaged peak grey levels of puncta.

### Behavioral analyses

The movement of single animals was digitally recorded at 30 frames per second [13]. A custom-written software automatically recognizes worm images from each frame and assigns thirteen points spaced equally from the head to the tail along the midline of the body. Supplementary angles were calculated from three consecutive points of thirteen points, and plotted along the body positions (11 total). Data were obtained when the head of an animal was maximally swung to the ventral side. To measure the speed of multiple animals, animals with the same genotype were corralled inside a copper ring [10], and their movement was recorded and analyzed with the image processing software Image J with plugin Mtrack2.

### Oocyte expression and electrophysiology

We injected cRNA encoding SLO-1a, the predominant splice variant of the *slo-1* gene [15], alone or together with cRNA encoding *islo-1_54-end_* (5 ng of each cRNA). Oocytes were cultured for 4 to 10 days at 18°C in modified L-15 medium [37]. We did not measure the expression levels of SLO-1 and ISLO-1 proteins.

External recording solutions consisted of (in mM): KMeSO_4_ (140), KCl (6), KHEPES (20), pH 7.2. Solutions of defined Ca^2+^ concentration were made by adding CaCl_2_ (0.1 mM) and the following Ca^2+^ buffers: NTA (5 mM) for 50-60 µM Ca^2+^; HEDTA (5 mM) for 3-20 µM Ca^2+^; and EGTA (5 mM) for 2 µM Ca^2+^ solution. Free calcium was measured using an Orion Ca^2+^ electrode. The pipette solution consisted of (in mM): KMeSO_4_ (140), KCl (6), MgCl_2_ (2), KHEPES (20), pH 7.2. Recording electrodes were pulled from borosilicate glass (Sutter Instruments, OD = 1.5 mm), coated with wax, and pressure polished [38]. Electrode resistances were 1.1–1.7 MΩ.

Currents were measured in inside-out patches with symmetric K^+^ concentrations using a WPC-100 patch clamp amplifier (Bioscience Tools, San Diego, CA). To determine the voltage-dependence of channel gating, we applied 20 ms voltage pulses between –80 and 200 mV (in 10 mV increments) from a holding potential of –80 mV. Calcium sensitivity was determined by exposing excised patches to a series of buffered Ca^2+^ solutions. Maximum patch currents were between 2–6 nA in amplitude. Analog data were sampled at 100 kHz and filtered at 5 kHz using an ITC-16 analog-digital converter (Instrutech, Great Neck, NY) and HEKA Patchmaster v2.15 software. Conductance-voltage curves (*G-V*) were derived from steady-state currents, which were converted to conductance. All voltages were corrected for errors due to residual series resistance. *G-V* curves were fit to the Boltzmann equation G/G_max_ = 1/1 + exp((*V*-*V*_1/2_)*z*F/RT), where *V*_1/2_ is the voltage of half maximum conductance, *z* is the slope of the curve, and R,T and F have the usual meaning.

## Supporting Information

Figure S1 Body curvature analysis of *islo-1* alleles, *dys-1* and *dys-1*;*islo-1*. (A) Bending angles of *islo-1(eg978)*, *dys-1* and *dys-1;islo-1* mutant animals. (B) Representative photos of animals in the same posture. (C) Bending angles of other *islo-1* alleles and *islo-1;Ex[mCherry::islo-1]*. Asterisks represent significant difference between two groups and ns represents not significant difference.
(0.91 MB TIF)

Figure S2 The physical map of *islo-1*, and the operon structure of *islo-1*, *pmk-3*, *pmk-2*, and *pmk-1*.
(0.23 MB TIF)

Figure S3 SGCA-1 is localized in the dense bodies of body wall muscle. In the upper panels wild-type animals were stained with antibodies raised against MHC-A (myosin heavy chain A, green) and SGCA-1 (red). In lower panels, wild-type animals were stained with antibodies raised against vincullin (green) and SGCA-1 (red). Vincullin is localized to dense bodies and attachment plaques.
(5.01 MB TIF)

Text S1 Supplementary methods.
(0.03 MB DOC)

Video S1 A 20 second movie from three groups of animals with different genotypes. *egl-19(gf) (upper left)*, *slo-1(gf)* (upper right) and *egl-19(gf);slo-1(gf)* (lower).
(1.06 MB MOV)
