# Supplementary figures and images for: The Dystrophin Complex Controls BK Channel Localization and Muscle Activity in Caenorhabditis elegans
Source: PLoS Genet. 2009 Dec 18;5(12):e1000780. doi: 10.1371/journal.pgen.1000780 (PMC2788698; doi:10.1371/journal.pgen.1000780)

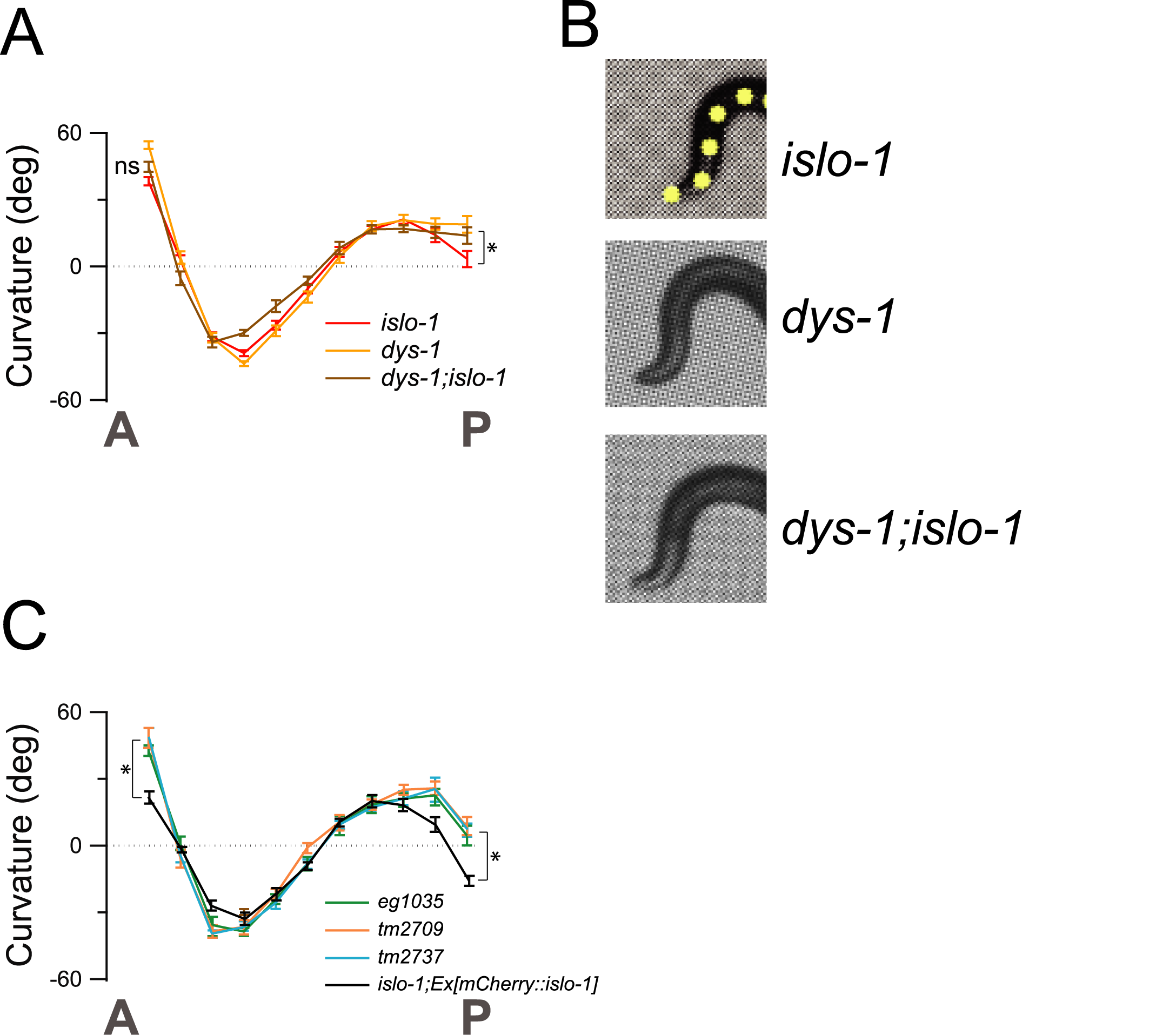

Supplement: Figure S1 — Body curvature analysis of islo-1 alleles, dys-1 and dys-1;islo-1. (A) Bending angles of islo-1(eg978), dys-1 and dys-1;islo-1 mutant animals. (B) Representative photos of animals in the same posture. (C) Bending angles of other islo-1 alleles and islo-1;Ex[mCherry::islo-1]. Asterisks represent significant difference between two groups and ns represents not significant difference. (0.91 MB TIF) [file pgen.1000780.s001.tif]

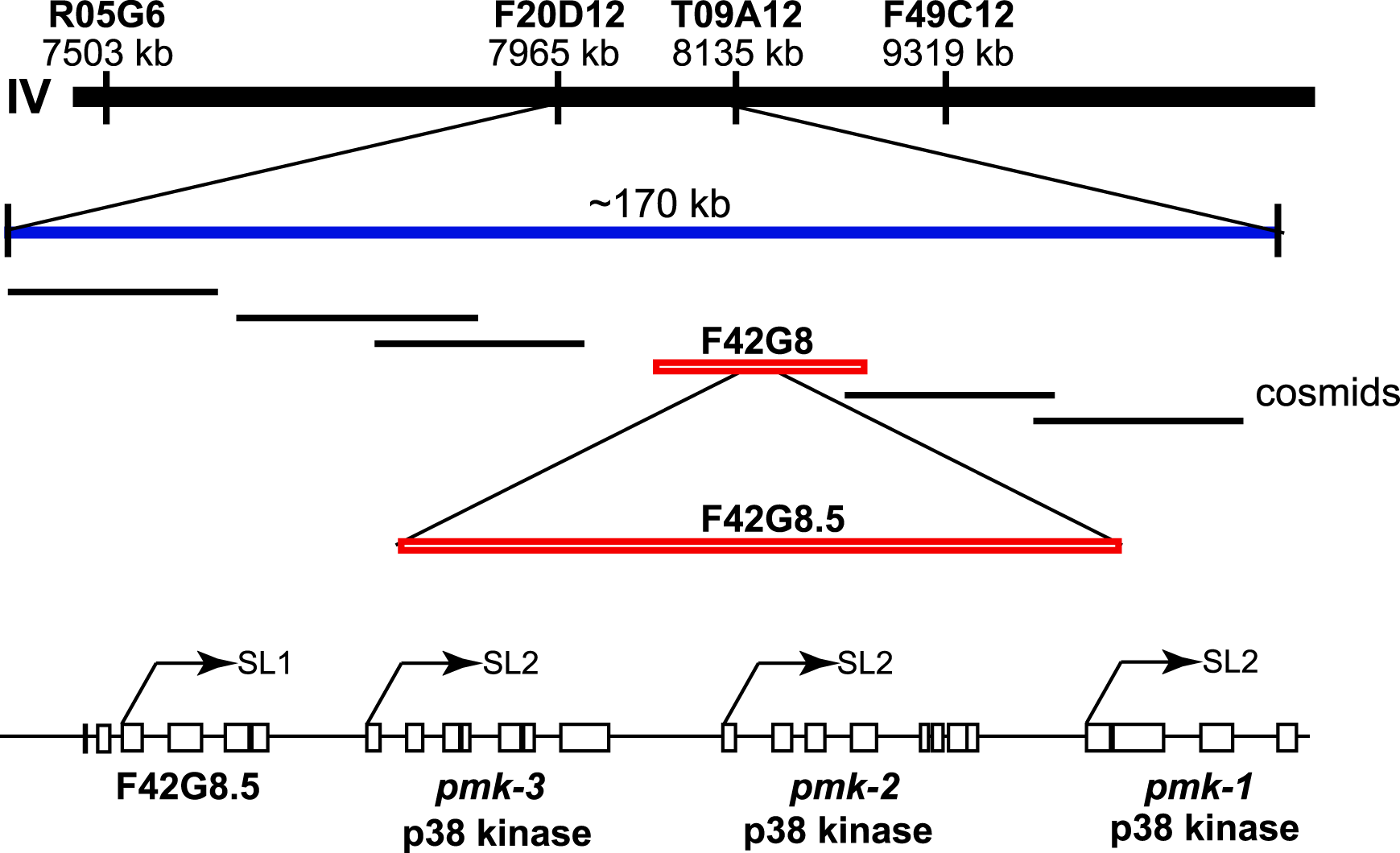

Supplement: Figure S2 — The physical map of islo-1, and the operon structure of islo-1, pmk-3, pmk-2, and pmk-1. (0.23 MB TIF) [file pgen.1000780.s002.tif]

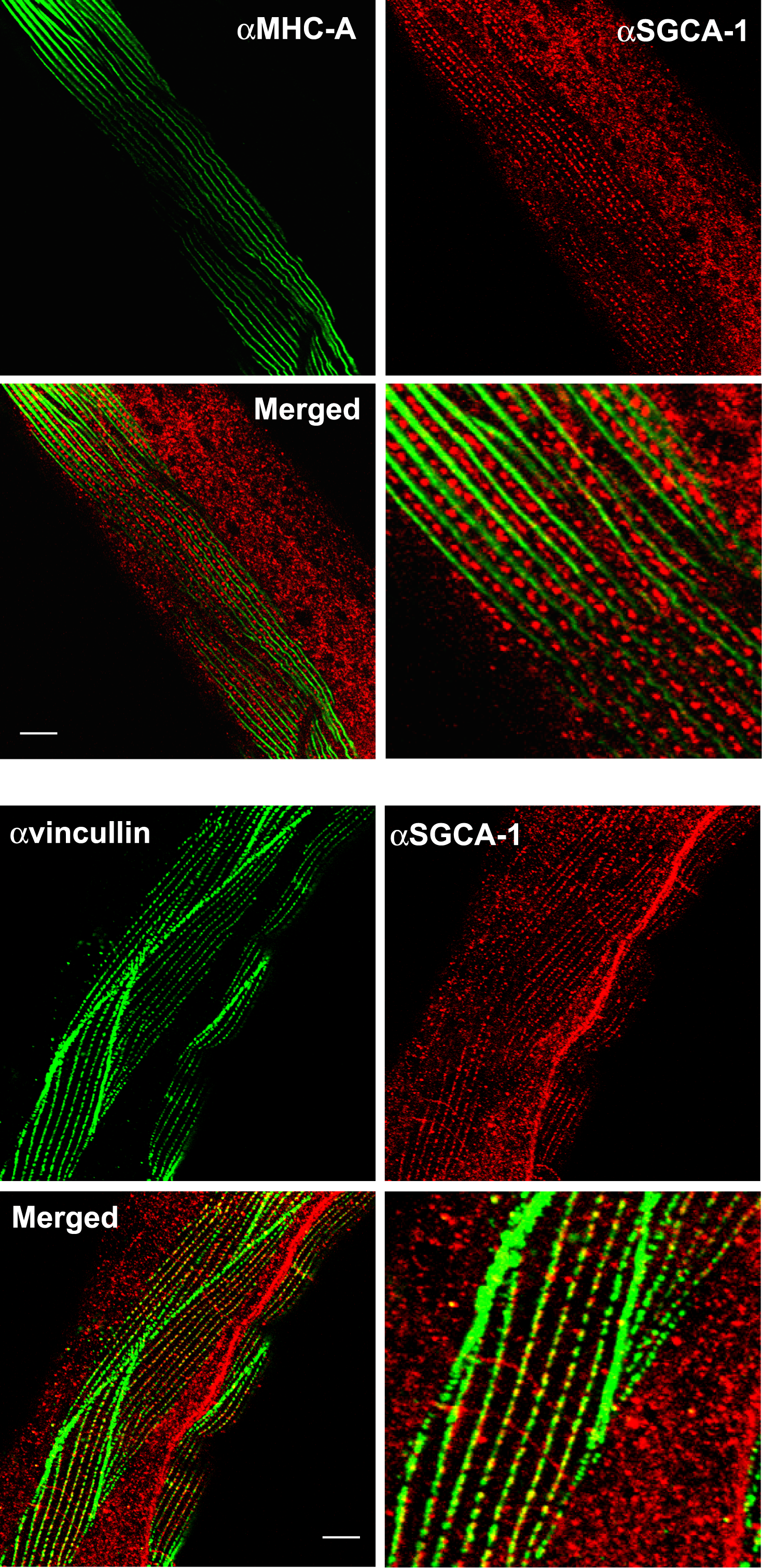

Supplement: Figure S3 — SGCA-1 is localized in the dense bodies of body wall muscle. In the upper panels wild-type animals were stained with antibodies raised against MHC-A (myosin heavy chain A, green) and SGCA-1 (red). In lower panels, wild-type animals were stained with antibodies raised against vincullin (green) and SGCA-1 (red). Vincullin is localized to dense bodies and attachment plaques. (5.01 MB TIF) [file pgen.1000780.s003.tif]
