# Supplementary material for: The Dystrophin Complex Controls BK Channel Localization and Muscle Activity in Caenorhabditis elegans
Source: PLoS Genet. 2009 Dec 18;5(12):e1000780. doi: 10.1371/journal.pgen.1000780 (PMC2788698; doi:10.1371/journal.pgen.1000780)
Supplement: Text S1 — Supplementary methods. (0.03 MB DOC) [file pgen.1000780.s004.doc]

**Text S1**

**Supplementary Methods**

**DNA constructs for transgenic animals**

To construct GFP:: *islo-1* or mCherry::*islo-1*, a PCR product amplified from the first exon of the upstream *isp-1* gene (which is transcribed in the other direction) to all of the introns and exons of *islo-1* (the entire *islo-1* gene and its promoter) was subcloned into the HindIII-EcoRI site of pPD118.20, and then GFP (or mCherry) cDNA was inserted to the third exon of *islo-1* gene in frame.

To construct *islo-1* or *islo-1*Δ4C, a PCR product amplified from the first exon of the upstream *isp-1* gene to all of the introns and exons of *islo-1* (the entire *islo-1* gene and its promoter) or to all of the introns and exons of *islo-1* lacking 12 nucleotides was directly used for generating transgenic animal in *islo-1* mutants.

P*_myo_*-*_3_*GFP::*islo-1_54-end_* was constructed by subcloning PCR-amplified *islo-1* partial cDNA (lacking 53 amino acid at the N terminus) into the EcoRI site of pPD118.20. Transgenic animals with this construct in *islo-1* do not exhibit the head-bending phenotype. For GFP::*sgca-1* construct, the GFP sequence was inserted in-frame right after the signal sequence of *sgca-1* open-reading frame. The resulting construct was injected to *sgca-1* mutants and rescued the head-bending phenotype.

For construction of *egl-19*::mCherry construct, we amplified the promoter (3.4 kb) and entire open reading frame of *egl-19* (12.3 kb) and then used an extended overlapping PCR reaction to attach the mCherry sequence to the end of *egl-19*. The PCR product was injected to *egl-19*(*st556*)/*unc-82*(*e1323*) *unc-24*(*e138*). Because the selected transgenic animals did not produce progeny with Unc phenotype, we concluded that this construct rescues the lethal phenotype of *egl-19*. Among these lines, we chose a transgenic line expressed mCherry at the lowest level for further analysis.

**Fixing and Immunostaining**

Young adult animals were permeabilized by three rounds of freezing and thawing, and then fixed in 2% paraformaldehyde, 80 mM KCl, 20 mM NaCl, 10 mM EGTA, 5 mM spermidine, 15 mM PIPES, pH 7.4, 25% methanol for 2 h at 4 °C. Cuticles of the animals were weakened by a reduction and oxidation reaction. First, the cuticles were reduced in Tris-Triton buffer (100 mM Tris-HCl, pH 7.4, 1% Triton X- 100, 1 mM EDTA) containing 1% β-mercaptoethanol for 1 h at 37 °C. Reduced cuticles were oxidized in borate buffer (33.3 mM boric acid, 16.6 mM NaOH, pH 9.5) containing 0.3% hydrogen peroxide for 15 min at room temperature. Processed samples were then treated with phosphate-buffered saline (PBS) containing 1% Triton X-100 and 10% goat serum at room temperature. Primary antibody was added to the sample and incubated overnight. The samples were then incubated with highly cross-adsorbed Alexa 594-conjugated goat secondary antibody (A11037, Invitrogen) was then incubated in PBS containing 10% goat serum at room temperature. We also confirmed that the secondary antibody does not cross react with any *C. elgeans* muscle components by staining a sample in parallel without adding primary antibody.

**Immunoprecipitation and Western blot**

Mammalian expression vectors were constructed with pCMV-Tag2B containing FLAG epitope sequence (Stratagene) or pHM6 containing HA epitope sequence (Roche) by sub-cloning *stn-1*, *slo-1a* or *islo-1_54-end_* cDNA. For co-immunoprecipitation of ISLO-1 and STN-1, ISLO-1 or ISLO-1Δ4C (lacking four amino acids at the C terminus) was tagged at the amino terminus with FLAG, while STN-1was tagged with an HA epitope. For co-immunoprecipitation of SLO-1 and ISLO-1, SLO-1 and ISLO-1 were tagged with FLAG and HA epitope, respectively. Different combinations of the constructs (a total of 8 micrograms) were transiently transfected into HEK293 cells grown in a 100 mm culture dish (Fugene 6, Roche). Forty eight hours later, transfected cells were lyzed with a modified RIPA buffer (50 mM Tris-HCl, pH 7.4, 1% NP-40, 0.5% CHAPSO or 0.25% deoxycholate, 1 mM NaVO_3_, 10 mM NaF, 10% glycerol, 150 mM NaCl). The resulting lysates were divided to two equal fractions and each fraction was subjected to immunoprecipitation with either anti-FLAG (M2, Sigma) or anti-HA antibodies (HA.11, Covance). Immune complexes were resolved in 10% SDS-PAGE and transferred to PVDF membranes (Bio-Rad). Membranes were cut off to yield the top and bottom membranes, and used for detecting immune reactions with either anti-HA or anti-FLAG antibodies.

**Behavioral analyses**

The crawling movement of single animals was digitally recorded at 30 frames per second (FLEA camera, Pt. Grey, Vancouver, Canada). A custom program written in ImagePro (Media Cybernetics, Bethesda, MD) automatically recognizes the worm from each video frame and calculates the positions of thirteen points spaced equally from the tip of the head to tail along the midline of the body. We limited analysis of video frames of positions where the head of the animal was swung maximally to the ventral side of the animal’s sinusoidal track, where the head-bending phenotype is most obvious. To quantify the head-bending phenotype, the values for the average angle formed by the three most anterior midline points was compared across genotypes. Qualitatively similar results were obtained by analysis of video frames where the animal’s head was swung maximally to the dorsal side of the track. To quantify tail-bending, the three most posterior midline points were used instead. At least twenty video frames obtained from seven to ten individual animals were used for each genotype. Average angle values were compared with planned two-tailed Student t-tests. To measure animal speed, the movement of separate groups of animals that were restrained within copper rings was digitally recorded at 5 frames per second for 1 minute (QICAM, Qimaging) and the average speed was derived by calculating the displacement of centroid positions from subsequent video frames with an image processing software (Image J, MTrack2 plugin).
